# Supplementary material for: CRISPR-based gene drives generate super-Mendelian inheritance in the disease vector Culex quinquefasciatus
Source: Nat Commun. 2023 Nov 20;14:7561. doi: 10.1038/s41467-023-41834-1 (PMC10662442; doi:10.1038/s41467-023-41834-1)
Supplement: Supplementary file 2 — Reporting Summary [file 41467_2023_41834_MOESM2_ESM.pdf]

nature portfolio

Corresponding author(s): Valentino M. Gantz

Last updated by author(s): Sep 5, 2023

Reporting Summary

Nature Portfolio wishes to improve the reproducibility of the work that we publish. This form provides structure for consistency and transparency in reporting. For further information on Nature Portfolio policies, see our Editorial Policies and the Editorial Policy Checklist.

Statistics

For all statistical analyses, confirm that the following items are present in the figure legend, table legend, main text, or Methods section.

n/a	Confirmed	
☐	☒	The exact sample size (*n*) for each experimental group/condition, given as a discrete number and unit of measurement
☐	☒	A statement on whether measurements were taken from distinct samples or whether the same sample was measured repeatedly
☐	☒	The statistical test(s) used AND whether they are one- or two-sided<br>*Only common tests should be described solely by name; describe more complex techniques in the Methods section.*
☐	☒	A description of all covariates tested
☐	☒	A description of any assumptions or corrections, such as tests of normality and adjustment for multiple comparisons
☐	☒	A full description of the statistical parameters including central tendency (e.g. means) or other basic estimates (e.g. regression coefficient) AND variation (e.g. standard deviation) or associated estimates of uncertainty (e.g. confidence intervals)
☐	☒	For null hypothesis testing, the test statistic (e.g. *F*, *t*, *r*) with confidence intervals, effect sizes, degrees of freedom and *P* value noted<br>*Give P values as exact values whenever suitable.*
☒	☐	For Bayesian analysis, information on the choice of priors and Markov chain Monte Carlo settings
☐	☒	For hierarchical and complex designs, identification of the appropriate level for tests and full reporting of outcomes
☒	☐	Estimates of effect sizes (e.g. Cohen's *d*, Pearson's *r*), indicating how they were calculated

Our web collection on statistics for biologists contains articles on many of the points above.

Software and code

Policy information about availability of computer code

Data collection	No Software or Code was used during the data collection.
Data analysis	The experimental data and the analyses that support the findings of this study are available in Zenodo with the identifier [https://doi.org/10.5281/zenodo.8005545]

For manuscripts utilizing custom algorithms or software that are central to the research but not yet described in published literature, software must be made available to editors and reviewers. We strongly encourage code deposition in a community repository (e.g. GitHub). See the Nature Portfolio guidelines for submitting code & software for further information.

Data

Policy information about availability of data

All manuscripts must include a data availability statement. This statement should provide the following information, where applicable:

- Accession codes, unique identifiers, or web links for publicly available datasets
- A description of any restrictions on data availability
- For clinical datasets or third party data, please ensure that the statement adheres to our policy

The plasmid sequences of the constructs generated in this manuscript are available in GenBank database under accession number NW925705 [https://www.ncbi.nlm.nih.gov/nuccore/NW925705], OR459947 [https://www.ncbi.nlm.nih.gov/nuccore/OR459947], and MW417419 [https://www.ncbi.nlm.nih.gov/nuccore/MW417419]. Additional information is provided in the Supplementary Information. All source data are provided along with this manuscript. They cover the raw phenotypical scoring data collected in the transgenesis and gene drive experiments, which are reported in the Supplementary Data 1-4 files in Microsoft Excel

format (.xlsx). All other data and information is available upon request from the authors.

Research involving human participants, their data, or biological material

Policy information about studies with human participants or human data. See also policy information about sex, gender (identity/presentation), and sexual orientation and race, ethnicity and racism.

Reporting on sex and gender	Not applicable to the research described in this manuscript as no Human subjects were involved.
Reporting on race, ethnicity, or other socially relevant groupings	Not applicable to the research described in this manuscript as no Human subjects were involved.
Population characteristics	Not applicable to the research described in this manuscript as no Human subjects were involved.
Recruitment	Not applicable to the research described in this manuscript as no Human subjects were involved.
Ethics oversight	Not applicable to the research described in this manuscript as no Human subjects were involved.

Note that full information on the approval of the study protocol must also be provided in the manuscript.

Field-specific reporting

Please select the one below that is the best fit for your research. If you are not sure, read the appropriate sections before making your selection.

[x] Life sciences [ ] Behavioural & social sciences [ ] Ecological, evolutionary & environmental sciences

For a reference copy of the document with all sections, see nature.com/documents/nr-reporting-summary-flat.pdf

Life sciences study design

All studies must disclose on these points even when the disclosure is negative.

Sample size	For Gene drive experiments, in our previous experience of similar analysis of gene drive effect using single fly pair crosses a number size of >8 is usually representative, describing the overall behavior. As much as possible we have attempted to meet this standards with mosquitoes, although the technical challenges of handling Culex quinquefasciatus, we did not meet this standard and performed experiments with N= 4 or N=7. Analyses of inheritance ratios were carried out using R version 4.1.3 (R Development Core Team). Estimated means and 95% confidence intervals were calculated by a generalized linear mixed model, with a binomial ('logit' link) error distribution fitted using the glmmTMB package. this analysis keeps in consideration the overall sample size and uses this information to better estimate the mean and its 95% confidence interval.
Data exclusions	No data was excluded.
Replication	To support our proof-of concept of gene drive in Culex mosquitoes, we performed two replicates for the gene white, evaluating drive with different genomic arrangements. Similarly, for kmo, we perform drive analysis using different inheritance modes of Cas9. Each gene replicate was successful.
Randomization	F1 mosquitoes were randomly collected from different F0 crosses to perform F1 crosses. experimental and Control groups are determined by the mode of inheritance and the transgenes carried in a deterministic fashion.
Blinding	For all the mosquito experiments performed we have analyzed fluorescence presence in the eyes of fruit flies or whole body for the mosquitoes. This type of scoring does not need the investigators to be blind as the evaluation of the phenotype is presence or absence of the fluorescent marker and there is no much room for interpretation that could be subjective.

Reporting for specific materials, systems and methods

We require information from authors about some types of materials, experimental systems and methods used in many studies. Here, indicate whether each material, system or method listed is relevant to your study. If you are not sure if a list item applies to your research, read the appropriate section before selecting a response.

Materials & experimental systems

n/a	Involved in the study
☒	☐ Antibodies
☒	☐ Eukaryotic cell lines
☒	☐ Palaeontology and archaeology
☐	☒ Animals and other organisms
☒	☐ Clinical data
☒	☐ Dual use research of concern
☒	☐ Plants

Methods

n/a	Involved in the study
☒	☐ ChIP-seq
☒	☐ Flow cytometry
☒	☐ MRI-based neuroimaging

Animals and other research organisms

Policy information about studies involving animals; ARRIVE guidelines recommended for reporting animal research, and Sex and Gender in Research

Laboratory animals	All white gene-drive and Cas9 line Culex quinquefasciatus transgenic animals were generated or assembled in a wildtype Culex quinquefasciatus (California) strain, a kind gift provided by Anton Cornell (UC Davis), named as the CA-wt line. All kmo gene-drive Culex quinquefasciatus transgenic animals were generated or assembled in a wild-type individuals used in these experiments originated from the TPRI (Tropical Pesticides Research Institute) strain. Age of animals used was comparable in each experiment and usually within few days from pupae eclosion.
Wild animals	Study did not involve wild animals.
Reporting on sex	We have analyzed separately the gene drive effect in both the male and female germlines, indicating whether differences were observed or not.
Field-collected samples	Study did not involve field-collected samples.
Ethics oversight	Culex quinquefasciatus are invertebrate animals and are exempt from IACUC oversight. UCSD: All maintenance and experiments were performed in a high-security Arthropod Containment Level 2 (ACL2) barrier facility. Pirbright Institute: All experiments were conducted at The Pirbright Institute IS4L arthropod containment facility under the necessary safety regulations for gene drive research.

Note that full information on the approval of the study protocol must also be provided in the manuscript.
