# Supplementary material for: CRISPR-based gene drives generate super-Mendelian inheritance in the disease vector Culex quinquefasciatus
Source: Nat Commun. 2023 Nov 20;14:7561. doi: 10.1038/s41467-023-41834-1 (PMC10662442; doi:10.1038/s41467-023-41834-1)
Supplement: Supplementary file 4 — Description of Additional Supplementary Files [file 41467_2023_41834_MOESM4_ESM.pdf]

Title: Supplementary Data 1 - The *w*6-gRNA drive injection and transgenesis counting data

Description: This table contains the raw counting data of G1 with eGFP positive(GFP) phenotype indicating the transgenesis occurrence. All hatched G0s were divided into male and female pools and crossed with wild-type individuals. Egg rafts from each pool were hatched and counted together.

Title: Supplementary Data 2 - The establishment of *w*6-gRNA transgenic line

Description: The recovered eGFP+/DsRed transgenic male was mated to wild-type females to establish a transgenic line. The G1s were divided and scored by different sex and fluorescent markers, and the data is reported in the table. The inheritance ratio of the w6-gRNA drive element was calculated by dividing the number of individuals carrying eGFP positive (GFP) by the total number of G1s.

Title: Supplementary Data 3 - The *white* locus GD data

Description: Raw counting data of the G2 progeny with phenotypic scoring for DsRed positive (DsRed), eGFP positive (eGFP), *white-*/*white-*, or no fluorescence (none). The Cas9 transgene was tracked by DsRed presence and gRNA transgene was tracked by eGFP presence. Transgene inheritance rates in G2 for each single-pair cross (marked as “G1 (single-pair) cross” in the table) were calculated. Average inheritance and standard deviation were calculated for each transgene as well. The data is subdivided into the following tabs:

1. Fig. 2c - **The male copying data**: Counting data and inheritance rates with the *w*6-gRNA derived from G1 male germlines.
2. Fig. 2d - **The female copying data:** Counting data and inheritance rates with the *w*6-gRNA derived from G1 female germlines.
3. Fig. 3b,c - **Marked chromosome w4 homing data:** Counting data, average inheritance, and conversion rates with a marked chromosome approach.

Title: Supplementary Data 4 - The *kmo* locus GD data

Description: Raw counting data of the G2 progeny with phenotypic scoring for Cas9 only (*Opie2*-DsRed), Cas9 and *kmo*-gRNA (*Hr5IE1*-DsRed) together, *kmo*-gRNA (*Hr5IE1*-DsRed) only, or wildtype (no fluorescence). The Cas9 transgene was tracked by *Opie2*-DsRed presence and *kmo*-gRNA transgene was tracked by *Hr5IE1*-DsRed presence which can be phenotypically distinguished (Supplementary Figure 3). Transgene inheritance rates in G2 were calculated. Average inheritance and standard deviation were calculated for each transgene as well.
